# Future Leaders to Watch – Christopher Toepfer and Manuel Schmid

**DOI:** 10.1242/bio.058568

**Published:** 2021-02-15

## Abstract

Future Leaders to Watch is a series of interviews with the first authors of a selection of papers published in Biology Open, helping early-career researchers promote themselves alongside their papers. Christopher Toepfer and Manuel Schmid are co-first authors on ‘Myosin super relaxation (SRX) a perspective on fundamental biology, human disease, and cardiac therapeutics’, published in BiO. Christopher is a Sir Henry Wellcome Postdoctoral Fellow and British Heart Foundation Centre of Research Excellence (CRE) Intermediate Transition Fellow in the department of Cardiovascular Medicine at the Radcliffe Department of Medicine, University of Oxford, UK. Manuel is a Research Assistant in the department of Cardiovascular Medicine at the Radcliffe Department of Medicine, University of Oxford, UK. They are investigating the mechanisms that control heart function and impact acquired and inherited cardiovascular conditions.


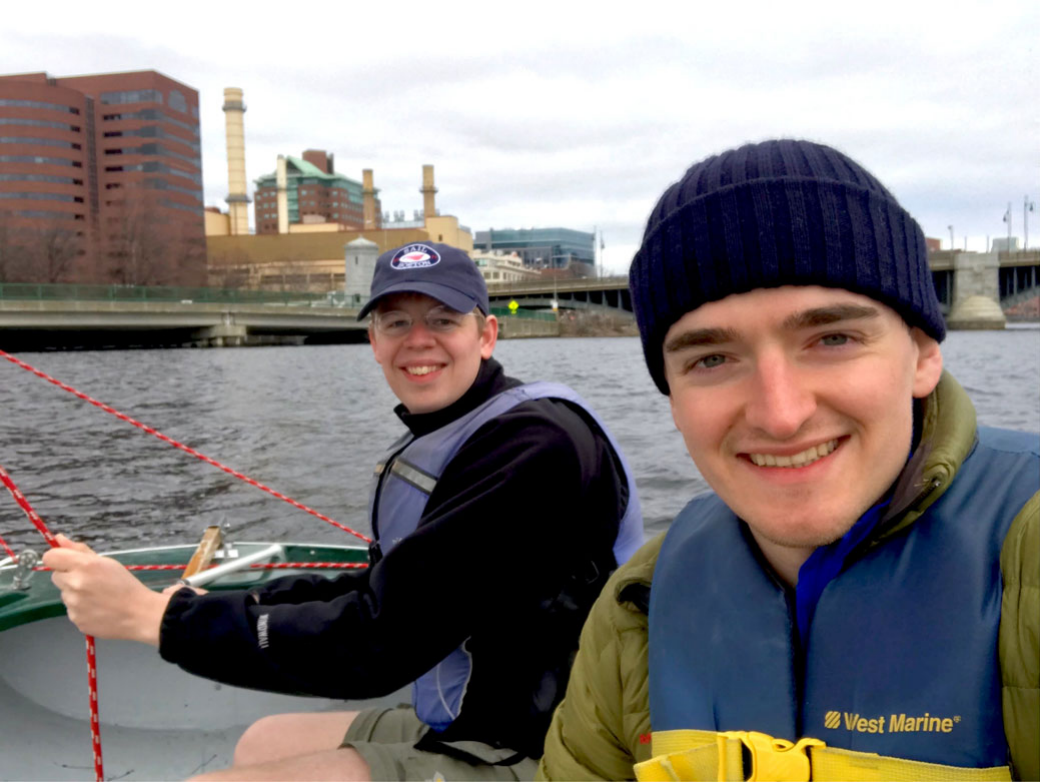


**Christopher Toepfer and Manuel Schmid**

**What is your scientific background and the story of how you got to where you are today?**

**CT:** I had a fascination with the heart from early in my career. As an undergraduate at Imperial College London, I chose to take the final year of my BSc in Biomedical Sciences as a focused year on cardiovascular biology. I had the opportunity to work over the summer in the laboratory of Professor Michael Ferenczi working on isolated muscle mechanics. At the end of my BSc I was awarded a Wellcome/NIH PhD studentship that then allowed me to carry on and expand these interests studying between Professor Ferenczi (Imperial College London) and Dr James Sellers (NHLBI, NIH) laboratories. In 2015 I took up a postdoctoral position with Professors Christine and Jonathan Seidman in Boston and evolved my interests into genetics, iPSC technologies and CRISPR/Cas-9 engineering. With the support of a Sir Henry Wellcome Postdoctoral fellowship I was able to continue my training with the Seidman laboratory and Professor Hugh Watkins at Oxford. During this time I have been able to focus on understanding cellular and protein level mechanisms that drive hypertrophic and dilated cardiomyopathies. As of late 2019, I moved to the University of Oxford to begin nucleating a group, focusing on understanding key mechanisms of contractile regulation in a wide variety of cardiovascular illnesses.

**MS:** Since I have always found myself drawn to various facets of medicine as a student, my academic education has long been two-pronged. In parallel to the pursuit of my medical studies, I started doing research in my third year at Medical school. In the context of a clinical traineeship at the Department of Paediatric Cardiology and Congenital Heart Defects at the German Heart Centre in Munich, I started to get more interested in this field. Under the mentorship of Assistant Professor Cordula Wolf and Professor Peter Ewert I had the opportunity to work on research projects and eventually join the Seidman Laboratory in Boston where I could lay the foundation for my medical doctoral thesis with support of the German Academic Scholarship Foundation. Professor Christine Seidman and Professor Jonathan Seidman enabled me to learn and work with an inspiring team of scientists focusing on genetic causes of cardiac diseases and potential ways of developing targeted therapies for them. In particular I have been working on regulation of protein expression by CRISPR/Cas9 engineering and its potential impact for future medical treatment. In this way I have also got to know my now supervisor and co-author Christopher Toepfer whose group at the Radcliffe Department of Cardiovascular Medicine I have joined last year after graduating from Medical School at the Technical University of Munich. There I am able to gain new insights and experience in the wide field of cardiac muscle physiology.

“There are new findings relating to how myosin super relaxation affects disease on a weekly basis.”

**What is the most important take-home message of your review?**

Controlling and regulating how the muscle of the heart beats is very important for maintaining the health of your heart throughout your life. This is especially true as the heart cells that we are born with are not replenished during our lives and must beat billions of times in the average lifespan. The processes that govern how energy is converted into contraction of muscle are tightly controlled, especially in the heart, and diseases that disturb this balance are detrimental to cardiovascular health. We do not know all of the mechanisms that alter muscle contraction in the heart, and we certainly do not yet know fully how they become disturbed in disease. Finding answers to these questions will be very important to understand heart disease and find novel treatments for it.

**What has surprised you the most while researching this review?**

**CT:** How quickly the field is growing and expanding our knowledge in this area. There are new findings relating to how myosin super relaxation affects disease on a weekly basis. This is really encouraging as this will invariably lead to better treatments for a wide variety of cardiovascular illnesses.

**MS:** Besides having the opportunity to realize how widespread the puzzle pieces of knowledge about myosin super relaxation already are, the review also revealed to me the promising new ground for future research and ideas to improve treatments for a wide amount of medical conditions affecting many patients in every age group and gender.

**Figure:**
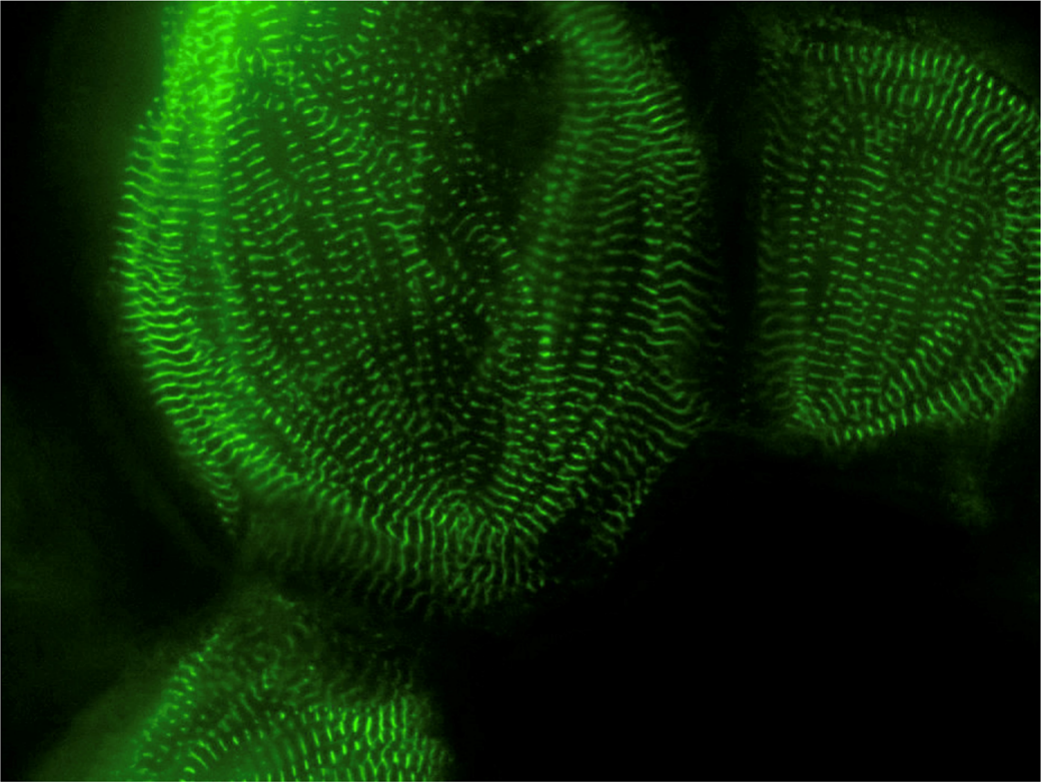
**A human induced pluripotent stem cell derived cardiomyocyte that has a genetically encoded GFP tag on the protein Titin.** We use this tag to quantify contractile function in these immature cell lines to model inherited heart conditions. (Featured in Sharma et al., 2018 and Toepfer et al., 2019).

**What do you feel is the most important question that needs to be answered to move the field forward?**

Myosin super relaxation (SRX) is only just beginning to be understood in the context of cardiovascular disease. There is still much to be understood about how it is regulated and controlled in healthy cardiac biology. Understanding these key mechanisms in the heart will invariably lead to breakthroughs in treatments for a wide variety of diseases. Additionally we really don't know how this state of myosin is affected in a wide variety of acquired cardiovascular conditions, which include myocardial infarction, hypertension, atrial fibrillation, and non-genetic heart failure.

“Understanding these key mechanisms in the heart will invariably lead to breakthroughs in treatments for a wide variety of diseases.”

**What changes do you think could improve the professional lives of early-career researchers?**

**CT:** I don't think this is a one glove fits all situation and depends largely on the individual. For this reason I believe strong and diverse mentorship is key. This empowers the researcher to overcome hurdles themselves, allowing them to advance their projects, interests, and career trajectory. I formed relationships with mentors even as an undergraduate and these people have been incredibly influential in my career and progress. Many of these people I still frequently speak to for advice.

**MS:** As a young researcher with a more clinical background I have always considered myself extremely fortunate having great mentors and supervisors who supported me with my first steps in basic science and reach out to me when needed. In the course of this, from my perspective support has been related to both specific help in scientific issues as well as defining someone's future visions and plans in the field. Therefor in my view a personal mentorship is crucial for early career-researches to establish themselves as well as to scientifically think out of the box (or should I say to look over the rim of the teacup, as a freshman in the UK).

**What's next for you?**

**CT:** We are excited to get projects up and running in the lab! We have multiple really exciting ideas about how to approach investigating key disease mechanisms in inherited cardiovascular conditions. The group is growing and it is a really fun period to enjoy with the other group members.

**MS:** I got to be part of a recently formed and highly motivated group, which will be carrying on its investigations to understand cardiac disorders and the functional basis of them. It is also a pleasure to collaborate with colleagues from Boston and Munich and to bring together knowledge and experience. I hope to be able to contribute to this way and also to keep being open to what medical science has to offer.
